# Midwives’ experiences and perceptions on the use of the Labour Care Guide: A qualitative study

**DOI:** 10.4102/curationis.v49i1.2798

**Published:** 2026-03-24

**Authors:** Nestor Tomas, Annarosa Poroto

**Affiliations:** 1 Department of General Nursing Science, Faculty of Health Sciences and Veterinary Medicine, University of Namibia, Rundu, Namibia

**Keywords:** Namibia, hospital, infant and newborn, midwifery, pregnancy

## Abstract

**Background:**

Over one-third of maternal deaths, nearly half of stillbirths, and a quarter of neonatal deaths are attributed to complications during labour and childbirth. Currently, the Labour Care Guide (LCG) is the only tool that promotes the implementation of the World Health Organization’s recommendations on intrapartum care, ensuring a positive childbirth experience. Previous studies examining midwives’ experiences in utilising the LCG did not include Namibia, hence this study.

**Objectives:**

This study aimed to explore and describe midwives’ experiences and perceptions of using the LCG at a training hospital in Namibia.

**Method:**

An exploratory descriptive qualitative design was employed to collect data from 10 midwives between May 2023 and July 2023. The interviews were audio-recorded, transcribed, and analysed using inductive reflective thematic analysis. The study adhered to the Consolidated Criteria for Reporting Qualitative Research reporting guideline.

**Results:**

The study identified three themes: LCG optimisation challenges, perceived usefulness of LCG and suggestions for improvement. While midwives expressed overall satisfaction with the new components of the LCG, some midwives perceived a shortcoming with regard to monitoring and managing labour progress in mothers who consumed traditional oxytocin known as Sivatu as being at risk for uterine rupture, foetal distress, or even death. The lack of resources and cultural norms on labour companionship poses LCG implementation challenges.

**Conclusion:**

This study found that while some midwives embraced the use of the LCG for monitoring labour and identifying abnormalities and complications, others perceived a limitation pertaining to monitoring women using traditional oxytocin.

**Contribution:**

The study thus recommends exploring alternative methods and strategies for safe labour monitoring and management.

## Introduction

Over one-third of maternal deaths, nearly half of stillbirths, and a quarter of neonatal deaths are caused by complications during active labour (World Health Organization [WHO] 2020). While some maternal and neonatal deaths could be averted through prompt and effective interventions (WHO 2020), about 94% of these fatalities occur during the use of the partograph in low- and middle-income nations. The partograph is a monitoring tool used to monitor the well-being of the mother and foetus during labour by displaying the progression of cervical dilation over time (Mugyenyi et al. 2024). The partograph has been utilised as a standard tool for monitoring women in the latent phase (Mugyenyi et al. 2024). However, it does not incorporate any provisions for midwives to administer mild pain relief medications, such as paracetamol. Recent evidence (Hofmeyr et al. 2021; Patabendige, Wickramasooriya & Dasanayake 2021) suggests that the partograph may not yield significant benefits. Moreover, current research and global priorities indicate that the partograph has not fulfilled its original goal of enhancing the childbirth experience (Hofmeyr et al. 2021; Patabendige et al. 2021; WHO 2018). This study aims to understand midwives’ experiences and perceptions of implementing WHO Labour Care Guide (LCG).

In 2018, the WHO, in accordance with new recommendations on intrapartum care to enhance the childbirth experience, conducted a review and revision of the partograph design (Patabendige et al. 2021; Vogel et al. 2021; WHO 2018). This led to the creation of the LCG, the next generation partograph, in response to emerging evidence (Pingray et al. 2021). On the other hand, the LCG is a comprehensive resource designed to facilitate the provision of high-quality, evidence-based, and compassionate care throughout the process of labour and childbirth, irrespective of the healthcare setting or level of service available (WHO 2020). When utilised appropriately, the LCG not only facilitates the accurate identification of prolonged labour promptly, thereby avoiding unnecessary procedures and interventions, but it also enables the timely implementation of interventions to mitigate the risks associated with obstructed labour, including postpartum haemorrhage, sepsis, uterine rupture, and maternal and neonatal mortality (Mugyenyi et al. 2024). Importantly, the LCG covers both the first and second stages of labour in a multifaceted way, with special attention paid to monitoring and avoiding prolonged labour, unnecessary oxytocin augmentation, and cesarean deliveries (Patabendige et al. 2021).

The implementation of the LCG allows healthcare practitioners to effectively observe the health status of expectant mothers and their infants throughout the childbirth process through routine evaluations designed to detect any abnormalities without unnecessary interventions (Ghulaxe et al. 2022; Henderson 2020; WHO 2020). In addition, the LCG includes the woman’s companion of choice during labour and childbirth, ensuring respectful care, good communication, confidentiality and the ability for women to make decisions about their pain management, labour positions, and pushing (WHO 2018). The implementation of the LCG has been found by many studies (Patabendige et al. 2021; Mugyenyi et al. 2024; Vogel et al. 2021) to provide evidence-based care, yet the evidence regarding its effectiveness remains inconclusive. While a study by Laisser et al. (2021) presented positive perspectives on the implementation of the LCG, Vogel et al. (2021) highlighted challenges in incorporating the companionship element of LCG in settings such as India, Kenya, Malawi, Nigeria, and Tanzania, primarily because of the substantial workload in those countries’ labour wards.

Patabendige et al. (2021) suggested that the introduction of the LCG should be a gradual process with continuous audits and quality assessment, while Bovbjerg, Pillai and Cheyney (2021) found the LCG to be an essential tool, but argued that more work needs to be done to address the root causes of maternal and infant morbidity and mortality. More extensive research thus needs to be done on context-specific experiences and unique challenges in order to provide tailored recommendations and insights that can enhance the overall effectiveness of the LCG.

The training of registered and enrolled midwives in Namibia includes undergraduate certificates, diplomas and degree programmes, as well as postgraduate education offering specialised courses such as an advanced diploma in midwifery and neonatal nursing care (The Government Republic of Namibia 2004). Although there is no substantial data available on the ratio of birthing women to midwives in Namibia, it is the responsibility of all midwives to oversee and facilitate deliveries (Mlambo & Amukugo 2023). The Ministry of Health recommends that all women give birth under the guidance of a midwife who possesses the necessary skills and knowledge to support them throughout the birthing process (Ashipala & Mutsindikwa 2022). However, the country is grappling with a shortage of essential equipment related to the birthing process and is struggling to address a 40% deficit in nursing staff, including midwives who serve as the primary point of contact for expectant mothers (Mbathera 2022; U.S. Agency for International Development 2019). Consequently, Namibia’s maternal mortality ratio is estimated to be between 215 and 265/100 000 live births (Ashipala & Mutsindikwa 2022; World Health Organization Labour Care Guide Research Prioritization Group 2023).The government of Namibia aims to reduce this ratio to 70/100,000 live births by 2030, in alignment with sustainable development goal 3 (SDG 3)-good health and well-being. However, an enquiry into maternal deaths in Namibia from 2018 to 2019 reported that, because of several challenges such as staff and equipment shortages, record keeping was not being done consistently (Heemelaar et al. 2023).

Midwives assume a diverse range of roles and carry out numerous responsibilities to guarantee high-quality care during the birth process (Mattison et al. 2020). To prioritise the safety and welfare of mothers and newborns throughout the childbirth process, Namibia recently introduced national guidelines for antenatal care services and maternity records (Ministry of Health and Social Services [MoHSS] 2021), and has replaced the partograph with the LCG since 2019. Midwives’ perceptions or beliefs of quality care serve as an indirect yet valuable indicator of the successful implementation of the WHO’s recommendations for intrapartum care in Namibia. Despite several studies on various labour practices aimed at improving outcomes in maternity care (Mugyenyi et al. 2024; Pingray et al. 2021; Vogel et al. 2021; WHO 2018), the only study (Laisser et al. 2021) that focused on midwives’ views of LCG revealed a reluctance to implement LCG. Although LCG presents numerous evidence-based benefits, including enhancements in the childbirth experience and improved outcomes when active labour begins at 5 cm of cervical dilation (Hofmeyr et al. 2021; Patabendige et al. 2021; WHO 2018), it does not accommodate women in the latent phase (5 cm), who necessitate some level of monitoring. Research in this area would yield valuable insights into midwives’ experiences and perceptions of implementing the LCG for monitoring expectant mothers in Namibia. This study explored and described midwives’ experiences and perceptions of using WHO LCG at a training hospital in Namibia.

## Research methods and design

### Study design

This study employed an exploratory descriptive qualitative research design to examine midwives’ perceptions of, and experiences with, LCG implementation at a training hospital in Namibia. An exploratory qualitative descriptive design was ideal to provide authentic, real-world expressions and a context-specific understanding (Doyle et al. 2020; Goins et al. 2021) of midwives’ experiences and perceptions regarding the implementation of LCG. The study followed the consolidated criteria for reporting qualitative research checklist (COREQ) to guarantee comprehensive documentation of the methods used (Tong, Sainsbury & Caraig 2007).

### Setting

The setting of this study was Rundu Intermediate Hospital, a public hospital in the eastern part of Namibia. The hospital is accredited by the Health Professions Council of Namibia as a training institution for health workers in Namibia. With a 300-bed capacity and over 500 medical employees, almost half of its employees are nurses and midwives (Tomas & Kachekele 2023). The departments are medical, surgical, obstetrics and gynaecology and emergency. The hospital is strategically located in Rundu, one of the fastest-growing towns in Namibia, given the Trans-Caprivi highway and its close proximity to Angola, Zambia, Zimbabwe and Botswana. The Rundu Intermediate Hospital was selected because it is the largest hospital in eastern Namibia and acts as a referral hospital for patients from Kavango East, Kavango West and the Zambezi region. The hospital recently built a state-of-the-art maternity section that caters for antenatal care, postnatal care and surgeries. It provides primary, secondary, and tertiary maternal healthcare services, and records over 1000 births per year (MoHSS 2021). Following the launch of new national guidelines for antenatal care services and maternity records by the MoHSS, Rundu Intermediate Hospital has since implemented the LCG to monitor the labour process (MoHSS 2021).

### Population and sampling

The study’s population consisted of midwives working in the maternity ward of a Namibian training hospital. A homogeneous purposive sampling technique was used to select 10 participants who shared a similar experience (Campbell et al. 2020) on the implementation of LCG. This number was deemed suitable based on literature that suggested that the sample size for achieving saturation ranges from 5 interviews to 24 interviews (Hennink & Kaiser 2022). It also made data analysis simpler, ensuring more meaningful findings. To be eligible, participants had to meet the following criteria: (1) registered or enrolled midwife; (2) working day and night shifts; and (3) working in the selected training hospital’s maternity ward. Day shift midwives typically work in a fast-paced environment with increased patient loads and enhanced interdisciplinary collaboration. Conversely, night shift midwives face distinctive challenges, such as reduced staff per shift which can affect the consistency of care. Nevertheless, the more tranquil ambience during the night presents an occasion for more individualised patient interactions, affording midwives the opportunity to offer more emotional support during a vulnerable period. Midwives who were on leave during the data collection phase were excluded from the study.

### Data collection

Data collection was conducted in May 2023 and July 2023 using a semi-structured interview guide to allow the midwives to best share their experiences and perceptions regarding the use of the LCG. The second author opted for this particular period in order to gather information before the conclusion of the academic year. A semi-structured interview guide was developed in accordance with the study’s objectives and in line with extant literature. A pilot study with four participants was conducted to assess and improve the clarity of the data collection instrument. The data from this pilot were not included in the main study. Open-ended questions were utilised to elicit comprehensive responses. The principal author approached potential participants at the hospital, explained the purpose and significance of the study, and requested their voluntary participation and consent.

To address any potential bias in participant recruitment and data collection, the study specifically targeted individuals who met the eligibility criteria. Furthermore, member checking was undertaken with all participants to verify the accuracy and truthfulness of the findings. Two key questions were posed: (1) Please share your experience and perceptions of using the LCG, and (2) What suggestions do you have for improving the LCG? Probing questions such as ‘Could you clarify?’, ‘What are your thoughts, and expectations?’, ‘What were your positive and negative experiences?’ and ‘Could you provide more detail?’ were also utilised to obtain more comprehensive information from the participants (see Table 1).

**TABLE 1:** Interview guide.

Interview questions
**Demographic related questions**
1. Gender
2. Age
3. Rank and position
4. Years of experience in maternity
5. Highest qualification
Main questions
6. Please share your experience and perceptions of using WHO Labour Care Guide (Probe: Positive and negative experiences views, and beliefs)
7. What suggestions do you have for improving the use of LCG?

LCG, Labour Care Guide; WHO, World Health Organization.

The interviews were recorded using an audio recording device and transcribed by the novice researcher, Annarosa Poroto, supervised by Nestor Tomas. The accuracy of the transcriptions was verified by the senior author with expertise in qualitative analysis. Recruitment was carried out until thematic or code saturation was achieved, resulting in a final sample size of 10 participants. As indicated by previous researchers (Braun & Clarke 2021; Hennink & Kaiser 2022), a lack of new codes or themes emerging from eight interviews suggests that code or thematic saturation has been achieved. Each interview lasted between 45 min and 55 min. The field notes and recordings were numbered from P1 to P10, according to the order of the interviews conducted.

### Data analysis

The data were transcribed verbatim through an inductive process. To identify patterns in the data, the six-step reflective thematic analysis method outlined by Braun and Clarke (2023) was employed. This method allowed for the researchers, to analyse the data using an organic approach and without using a structured coding process (Braun & Clarke 2023; Campbell et al. 2021). In order to familiarise with the data, the second author, listened to the recordings repeatedly and examined transcripts before they were entered into a Microsoft Word document. The researchers read and analysed data from various sources to generate initial codes, documenting their reflections and emerging ideas.

To generate initial themes, the second author sorted the different codes in a table, including documenting any ideas that came to mind. After going through the transcripts, the second author put similar codes together by highlighting them in different colours to make the analysis easier. Subsequently, the second author conducted a cross-verification of the codes prior to their integration with the observational notes and recordings. Both authors then clustered the similar codes together and labelled them into meaningful groups of subthemes, before reviewing them by going through the entire coded data set and assigning them to the corresponding segments of text. The data organisation was assessed to identify any new themes. To define and name the themes, the relationships between subthemes and themes were recognised to reduce the overall list. Grouping them together and indicating their relationships helped establish the connections. The themes and subthemes were subsequently structured under three final themes, namely: (1) LCG optimisation challenges; (2) perceived usefulness of the LCG and (3) suggestions for improvement.

### Methodological rigour

The study utilised the following quality criteria of trustworthiness: credibility, dependability, conformability and transferability (Lincoln & Guba 1985; Pratt, Sonenshein & Feldman 2022). Credibility was established through the use of purposive sampling methods, i.e. the participants met the inclusive criteria, and by recording the interviews to avoid losing the data (Campbell et al. 2020). Member checking was conducted through participants’ review and validation of the study’s findings. Following a thorough examination of the participants’ feedback, no discrepancies or concerns were identified. As a result, the initial findings were upheld without modification. As a basis for credibility, the research team, which was comprised of the novice researcher and a senior researcher, had experience in qualitative research who authored numerous qualitative papers in international peer review journals. Dependability was ensured through the use of recorded data and an inquiry audit trail, which was performed by the research supervisor to confirm the accuracy of the findings. The study ensured transferability by providing thick descriptions of the procedures used for data collection (Braun & Clarke 2021; Lincoln & Guba 1985; Younas et al. 2023). The data reported in this study were based on the study’s objectives and are representative of the participants’ experiences and perceptions of midwives on the implementation of the LCG in Namibia. Moreover, transferability was ensured by following the components of authenticity, meaningfulness, contextuality, emic perspective and linking them to participants’ experiences based on their understandings, while observing participants’ social and cultural beliefs, body expressions, quotes, and settings (Younas et al. 2023). To prevent premature data analysis, the researchers allocated sufficient time to thoroughly examine the data before conducting the analysis in manageable segments. This method helped to develop a comprehensive understanding of the contexts of the midwives’ experiences, thereby improving their coding decisions and avoiding the creation of superficial codes (Braun & Clarke 2021; Inayat et al. 2024). Furthermore, we acknowledge researcher subjectivity as an integral component of the research process, recognising the inherent interpretative nature of coding. Consequently, coding was not conceptualised as a pursuit of absolute accuracy, but rather as a process through which meaning, understood to be fluid and actively constructed, emerges from the data rather than being inherently fixed within it.

### Ethical considerations

The study was carried out with the approval of the School of Nursing and Public Health Ethical Committee at the University of Namibia (reference number: SoN 02/2023), and the MoHSS (reference number: 22/4/2/3), and all participants provided written informed consent before joining the study. The participants were also notified that they had the option to withdraw from the study at any point with no repercussions. The study’s confidentiality and anonymity were ensured by not collecting any personal identifying information during data collection. The researcher obtained written informed consent from each participant before conducting and recording the interviews. Only the researchers had access to the data collected, which were encrypted and password-protected before being destroyed after the transcriptions were complete. This study adhered to the revised Declaration of Helsinki, ensuring the protection of human participants through informed consent.

## Results

### Participant demographic characteristics

Ten midwives, aged between 26 and 46 years, were recruited for the study. Their work experience varied from 2 years to 16 years. Six participants held Bachelor’s Degrees in Nursing Science, two had Diplomas in Nursing Science, and the remaining two possessed Certificates in Nursing and Midwifery Education (see Table 2).

**TABLE 2:** Characteristics of participants.

Participant number	Gender	Age (years)	Working experience (years)	Rank and position	Education
P1	Female	46	16	Registered midwife	BSc in Nursing and Midwifery
P2	Female	38	16	Registered midwife	Diploma in Nursing and Midwifery
P3	Female	37	14	Registered midwife	BSc in Nursing and Midwifery
P4	Female	33	4	Registered midwife	BSc in Nursing and Midwifery
P5	Female	43	2	Registered midwife	BSc in Nursing and Midwifery
P6	Female	26	2	Registered midwife	BSc in Nursing and Midwifery
P7	Female	27	2	Registered midwife	BSc in Nursing and Midwifery
P8	Female	28	2	Registered midwife	Diploma in Nursing and Midwifery
P9	Female	30	10	Enrolled midwife	Certificate in Nursing and Midwifery
P10	Female	34	3	Enrolled midwife	Certificate in Nursing and Midwifery

### Presentation of the themes

The study identified three major themes: LCG optimisation challenges, perceived usefulness of the LCG and suggestions for improvement (see Table 3).

**TABLE 3:** Summary of findings.

Themes	Subthemes
1. LCG optimisation challenges	1.1. Challenges related to monitoring traditional oxytocin-induced labour 1.2. A diagnostic challenge 1.3. An imminent threat of a medical equipment shortage 1.4. Limited companionship
2. Perceived usefulness of LCG	2.1. Supportive care labour assistance 2.2. A comprehensive labour monitoring solution
3. Suggestions for improvement	3.1. Resource mobilisation 3.2. Consider adding the missing pieces

LCG, Labour Care Guide.

#### Theme 1: Labour care guide optimisation challenges

This theme consists of four subthemes, generated as the midwives narrated their experiences regarding challenges pertaining to the successful implementation of the LCG in monitoring labour processes at Rundu Intermediate Hospital in Namibia.

**Subtheme 1.1: Challenges related to monitoring traditional oxytocin-induced labour:** Challenges were described regarding monitoring labour progress in mothers who use traditional oxytocin, specifically in the Kavango East region. These midwives believe that the use of traditional oxytocin or Sivatu, made from elephant dung, often leads to fast labour, making it difficult to assess progression. Additionally, mothers who use Sivatu experience more intense contractions, which can result in spontaneous delivery:

‘The patient can come in 3 cm or 2 cm dilated. After 30 min, this patient is fully dilated. These mothers present with strong contractions that are spacing apart, and they deliver fast. Most of the time, it is due to the traditional dung they drink before coming to the hospital, which makes it difficult to review labour progress.’ (P3, 37 years, registered midwife)

‘We have traditional oxytocin, which can cause the patient to experience precipitated labour. Sometimes, we don’t even have the chance to perform a caesarean section because the patient’s cervix quickly dilates.’ (P5, 43 years, registered midwife)

Two midwives spoke about their challenges with the use of traditional oxytocin. They mentioned that although it can induce labour, the use of Sivatu also presents other risks to both the mother and baby, including uterine rupture, foetal distress, and even the death of babies:

‘We had cases of other mothers who ended up with uterine rupture, high rates of intrauterine deaths, antepartum haemorrhage, and cord prolapse.’ (P8, 28 years, registered midwife)

‘[*With a long face*] The contractions are very strong due to the traditional oxytocin they drinking, resulting in foetal distress. Some unlucky babies die, and some mothers develop high blood pressure and sepsis.’ (P9, 30 years, enrolled midwife)

One participant noted, however, that some mothers believe that traditional oxytocin is good for the well-being of their unborn baby:

‘[*Raised eyebrows*] The combination of Sivatu [*traditional oxytocin*] and elephant dung is believed to protect the baby.’ (P3, 37 years, registered midwife)

**Subtheme 1.2: A diagnostic challenge:** According to the midwives, it is important to monitor and care for mothers throughout every stage of labour, regardless of cervical dilation. Some of the midwives emphasised the challenges they encounter in diagnosing a prolonged latent phase of labour and identifying abnormalities early because of the absence of the alert and action line and small blocks on LCG:

‘The LCG is normally opened at 5 cm. Therefore, diagnosing prolonged labour becomes a challenge because there is no latent phase to indicate the onset of labour.’ (P1, 46 years, registered midwife)

‘The removal or exclusion of the latent phase of labour makes it challenging to detect abnormalities compared to its inclusion in the partograph. With the new LCG, labour starts from a dilation of 5 cm.’ (P7, 27 years, registered midwife)

Although, two midwives mentioned that women in the latent phase of labour require frequent monitoring, the exclusion of this phase from the LCG poses a challenge when it comes to diagnosing a prolonged latent phase:

‘LCG does not involve women in the latent phase of labour. As a result, they are not monitored as they should be. To compensate for this, we opted to monitor them every four hours, just like any other pregnant women in active labour.’ (P3, 37 years, registered midwife)

Another participant mentioned that midwives need to frequently review the patients’ files to diagnose prolonged labour. They do this by determining the dilation of the patient from the time of admission:

‘[*Pulling her hair back*] The exclusion of the latent phase on the LCG makes it difficult for us to detect a prolonged latent phase of labour. Therefore, to diagnose this, you have to check the woman’s file from the admission time and determine how long the patient was at 4 cm dilation.’ (P10, 34 years, enrolled midwife)

Some concerns were shared regarding the challenge in identifying the alert and action lines on the LCG, making it difficult for midwives to identify abnormalities by simply looking at it:

‘It is difficult to identify abnormalities unlike with the old partograph that had clear alert and action lines. With the old partograph, we could easily tell if our graph was on the alert line, which meant that the patient was in danger and action needed to be taken.’ (P7, 27 years, registered midwife)

Frequently, the midwives raised concerns about the size of the writing blocks on the LCG, arguing that they are too small for recording information, leading to the omission of some data:

‘[*Looking down*] The spacing of the recording is very small on the LCG, causing us to leave out important information. We are unable to record everything due to limited space. Additionally, some of us have large handwriting, making it difficult to fit everything in the provided space unless we summarise as much as possible.’ (P1, 46 years, registered midwife)

‘The spacing is quite small for some people with large handwriting, especially when it comes to writing our assessments and planning. Those blocks are very small and we have a lot of information to write there. The space may not be enough …’ (P6, 26 years, registered midwife)

Some midwives believed that the removal of the latent phase of labour from the LCG was actually beneficial. They argued that LCG helps prevent misdiagnosed prolonged latent phases and unnecessary medical interventions by initiating labour monitoring at 5cm dilation. Midwives noted that certain mothers, particularly first-time mothers, could remain in the latent phase of labour for a period of one day or even longer:

‘Personally, I feel it is much better that we start at 5 cm dilation and not during the latent phase, because with the latent phase, some patients, especially primigravidas, remain in the latent phase for a prolonged period before reaching the active phase of labour, therefore the LCG prevents misdiagnosing of the prolonged latent phase of labour.’ (P4, 33 years, registered midwife)

‘[*Rolling her eyes*] The latent phase varies from patient to patient and some may remain there for two days and we may end up with an unnecessary prolonged labour on the LCG, so it [*the latent phase of labour*] not being there is fine.’ (P5, 43 years, registered midwife)

**Subtheme 1.3: An imminent threat of medical equipment shortage:** In order to monitor labour progress effectively, appropriate medical equipment such as a Pinard stethoscope and cardiotocography (CTG) devices are essential. The midwives expressed that they had difficulties accessing such equipment, particularly foetal heart rate monitors, which can hinder their ability to make regular observations. Most of the midwives explained that their maternity wards are equipped with only one or two machines, which need to be shared among numerous patients. These machines are also used in various settings, including antenatal care rooms, admission rooms, and labour rooms:

‘[*I*]f you are monitoring a lot of patients at the same time, you need to have a lot of machines which we do not have. If we talk about the labour ward then we only have one machine, but monitoring a lot of patients at the same time makes it challenging.’ (P2, 38 years, registered midwife)

‘[*Distressed face*] There is a shortage of equipment, and sometimes these machines break easily and get damaged easily. So now the entire maternity ward has to share two or one equipment while there are already a lot of patients.’ (P3, 37 years, registered midwife)

Moreover, these machines were not only used to monitor women, but they were also used to print CTGs, especially for mothers who presented with bradycardia or tachycardia and those who were at high risk. Additionally, some machines freeze during use:

‘[*S*]ome patients are at high risk and they need continuous monitoring, especially patients with bradycardia or tachycardia. Then you have those who are on LCG monitoring. It becomes a challenge because the continuous monitoring machines cannot be stopped.’ (P1, 46 years, registered midwife)

‘[*Deep sigh*] On equipment, we have some challenges because some CTG machines are not functioning well. Some tend to freeze, while others may have missing or non-functioning probes. Additionally, some machines can stop working abruptly.’ (P6, 26 years, registered midwife)

**Subtheme 1.4: Limited companionship:** Support provided by birth companions during labour and childbirth improves maternal and neonatal outcomes and enhances a woman’s childbirth experience. Midwives in this study described negative experiences with the companion aspect in the LCG, however. While companionship was somewhat preferred, some midwives disputed its inclusion because of a lack of support for the practice in the hospital’s infrastructure. Additionally, companionship during the labour process is seen as a foreign concept that goes against the norms and values of many black-African communities in Namibia:

‘In our setting, companionship does not work, and we do not allow women to come here with their partners to give them any support they need. Therefore, this is one part of the labour care guide that is unnecessary.’ (P2, 38 years, registered midwife)

‘[*Faded smile*] We do not encourage companions during labour and delivery because of the structure of our maternity ward. The way the hospital is built does not allow it.’ (P3, 37 years, registered midwife)

Some midwives expressed distress over the impossibility of authorising a companion because of the congestion of their labour wards with patients, which could lead to chaos and confusion. The midwives believed that an overcrowded facility could result in a lack of privacy and confidentiality:

‘Our labour rooms do not have enough screens or space to accommodate an additional patient. This makes it difficult to allow companions to come, as it would lead to chaos, confusion, and a lack of privacy.’ (P7, 27 years, registered midwife)

‘[*Scowling*] Now imagine all women having companions. We usually have around 14 continuous deliveries throughout the day, so where can we keep their companions? And also, when it comes to privacy and confidentiality, how can we maintain it if they are all in one room?’ (P10, 34 years, enrolled midwife)

Moreover, one participant opposed the idea of allowing a supportive partner during labour and childbirth, arguing that it was a foreign concept and only suitable for Western countries. They believed it went against the norms and values of African people:

‘[*Stuttering*] The companion block on the LCG is not relevant to us because, when you look at it, a partner for an example is supposed to be present when the women starts labour. In most cases, we Africans do not allow such things. It’s not like people from Western countries who would request their partners to come support them.’ (P4, 33 years, registered midwife)

#### Theme 2: Perceived usefulness of Labour care guide

This theme focuses on the midwives’ views on the perceived usefulness of LCG in monitoring labour. Two subthemes were generated: supportive care labour assistance and a comprehensive labour monitoring solution.

**Subtheme 2.1: Supportive care labour assistance:** Evidence from this study shows that women who receive continuous support during labour are more likely to have spontaneous births, tolerate pain better and have shorter labours. Midwives also described positive experiences with the supportive care elements in the LCG, for example, they appreciated receiving oral fluids because they help keep the mother hydrated and provide the energy needed for pushing the baby out:

‘[*T*]he patient needs oral fluids, which are necessary for the patient to stay hydrated all the time. This helps with the progression of labour to be fast when the patient is well hydrated.’ (P1, 46 years, registered midwife)

In addition, the midwives acknowledged the effectiveness of pain relief during labour. However, they utilise non-pharmacological pain management techniques such as deep breathing exercises, back rubs, massages, and providing moral support to the mothers:

‘We prefer rubbing their back and teaching them deep breathing techniques. This is what we normally emphasise.’ (P10, 46 years, enrolled midwife)

‘[*W*]e teach patients deep breathing techniques and apply cold compressions to the mother’s back to relieve pain.’ (P6, 26 years, registered midwife)

The midwives are only allowed to give paracetamol for pain relief. In exceptional situations, other medications like morphine and pethidine are given with a doctor’s prescription, because of their potentially harmful effect on a baby:

‘For pain relief, we only administer paracetamol. However, if the doctor has prescribed drugs such as morphine, which are allowed to be administered to a patient during labour for pain relief, then you can administer them.’ (P8, 28 years, registered midwife)

‘We rarely administer pethidine or morphine as they usually cause drowsiness in the baby, so we generally avoid administering them and instead provide paracetamol to patients.’ (P10, 34 years, enrolled midwife)

**Subtheme 2.2: A comprehensive labour monitoring solution:** The LCG helps midwives monitor maternal and foetal well-being, track the progress of labour and identify potential complications. Some midwives in this study spoke positively about the LCG as a comprehensive labour monitoring tool, emphasising that the LCG is clear, includes most of the patient’s information, and is more advanced than the partograph. The tool also provides instructions to guide midwives in making decisions:

‘The LCG is more advanced compared to the previous one [*partograph*]. It is easy to use because there are full instructions on the LCG that guide you on what to record when we open it for a patient in active phase of labour.’ (P6, 26 years, registered midwife)

‘This LCG provides all the necessary information, making it helpful for users who may need a reminder of what is considered normal or abnormal.’ (P1, 46 years, registered midwife)

#### Theme 3: Suggestions for improvement

Participants in this study suggested their views on how to improve the use of the LCG. Two subthemes that emerged include resource mobilisation and adding the missing pieces on the LCG.

**Subtheme 3.1: Resource mobilisation:** Participants suggestions show how medical equipment and human resources play an important role in healthcare delivery. Participants in the study proposed the provision of human resources and medical equipment, such as foetal heart rate monitors to ensure a smooth monitoring of the LCG and printing of CTGs:

‘I am suggesting for the acquisition of more machine for smooth monitoring of patient in labour.’ (P1, 46 years, registered midwife)

‘[*I*]f they can recruit more staff then it would be fine, if there will be adequate staff then the workload will be shared and this will ensure that the woman gets the maximum care that they need.’ (P10, 34 years, enrolled midwife)

**Subtheme 3.2: Consider adding the missing pieces:** Participants suggested amending the LCG to include context-specific information, such as details on precipitated labour resulting from the use and consumption of traditional oxytocin. They explained this would help health workers accurately interpret labour progress directly from the LCG:

‘I think they should put a space for us to indicate if a patient took traditional oxytocin.’ (P9, 30 years, enrolled midwife)

‘In Kavango, we need a space [*column*] where we can record about traditional oxytocin called Sivato and elephant dung. So they should be column were its written that traditional oxytocin then we have to tick if patient took, on yes and if not, then on no because it always disturbs our LCG.’ (P3, 37 years, registered midwife)

Furthermore, participants suggested for the spacing on the LCG to be increased to enable them to have enough space for their observations:

‘They should just make it a bit larger for us to have enough space to write in.’ (P3, 37 years, registered midwife)

‘[*They should increase the spacing of the blocks*].’ (P10, 34 years, enrolled midwife)

## Discussion

In this study, we explored and described midwives’ experiences and perceptions of the use of the WHO’s LCG at a training hospital in Namibia. The findings revealed several barriers to utilising the LCG. Midwives highlighted that the use of traditional oxytocin (Sivatu) by some pregnant mothers inadvertently leads to accelerated labour, presenting challenges for midwives to effectively monitor the progression of labour using the LCG because of rapid delivery. Furthermore, apart from its role in precipitating spontaneous birth, Sivatu also poses potential risks that can be life-threatening for both the mother and the baby. These findings are in agreement with those of Makombe et al. (2023) and Haikera (2021), who discovered that the practice of using herbal medicine during pregnancy and in labour can result in adverse obstetric outcomes such as uterine rupture. Most midwives indicated that expectant mothers in Kavango East region consume traditional oxytocin, primarily because of their belief that it provides protection for their infants. This finding agrees with previous studies that reported that most women around in the world believe that utilisation of specific herbal medicine is beneficial in hastening labour duration and protecting a baby from harm (Kamel, Boullani & Cherrah 2022; Tsitsi, Tabona & Esther 2021). There is, however, limited evidence of the safety of traditional concoctions such as Sivatu on unborn babies. Future longitudinal and ethnographic studies are thus warranted to investigate the safety of these medications.

The study also found inconsistencies regarding the exclusion of the latent phase of labour from the LCG. Some midwives expressed concerns that the lack of clear alert and action lines makes it difficult to identify problems related to a prolonged latent phase, especially on busy days. The findings of this study support the conclusions of Laisser et al. (2021) and Vogel et al. (2021), who shared that the absence of a graphical display on a chart makes it challenging for practitioners to interpret and identify abnormal labour progress. According to Hofmeyr et al. (2021), the decision to exclude the latent phase of labour in the LCG was based on several factors, including that defining the latent phase was problematic because of its unclear onset and varying duration among women. Nonetheless, some midwives expressed their satisfaction with the absence of the latent phase in the LCG, citing this prevents misdiagnosis of a prolonged latent phase and unnecessary medical interventions, especially in primigravida women. These findings are as per Angeby (2018), who emphasised that removing the alert line is important to prevent unnecessary interventions, such as caesarean section deliveries, instrumental births and oxytocin augmentation. These interventions come with risks like uterine rupture and foetal distress, which can potentially harm both the mother and the baby. To address this, documentation of labour progress is initiated only at 5 cm dilatation once the active phase is diagnosed. Arguably, the discontinuation of the partograph might have provoked anxiety and even antipathy among some practitioners, hence the need to make resources available to support the implementation of the LCG. The lack of consensus regarding timing in monitoring the latent phase of labour could be because of considerable individual variability among patients (Cohen & Friedman 2023).

The midwives also raised concerns regarding the shortage and unavailability of certain essential equipment, such as foetal heart rate monitors. These concerns were exacerbated by a growing number of patients, leading to a suggestion for resource mobilisation. This finding is in alignment with Vogel et al. (2021), who highlighted the significance of ensuring the availability of crucial equipment during the implementation of the LCG. According to Housseine et al. (2020), maternity units in low- and lower middle-income countries are experiencing a growing issue of overcrowding and are facing a significant resource crisis. Under such circumstances, it is extremely challenging to adhere to the LCG’s surveillance regime while simultaneously caring for more than two women in a country like Namibia, which grapples with a scarcity of medical equipment (Kasuto 2022; Rasmeni 2022). These findings regarding insufficient monitoring of mothers during labour lead to delayed identification of problems and increased complications for both mothers and babies (Moyimane, Matlala & Kekana 2017). Equally, insufficient midwives being employed in the maternity ward was highlighted as a major challenge to effectively monitor women in labour. These findings emphasise the necessity for midwives to receive comprehensive training and have readily-available resources to proficiently and effectively utilise the LCG across diverse clinical settings (Mugyenyi et al. 2024; WHO 2023). Similarly, Lumadi, T.G. & Matlala, M.S. (2019) discovered that a shortage of midwives has a direct impact on the provision of quality care.

While companionship is often seen as a positive, midwives in this study surprisingly identified it as a barrier to implementing the LCG. They cited limited hospital rooms and certain African cultural beliefs as the primary reasons. They argued that allowing companions would compromise patients’ privacy and confidentiality and that it is a Western concept, as African men are not permitted to witness women giving birth. Even though companionship during labour is known to provide comfort and advocacy (Afulani et al. 2018; Singh et al. 2021), a key finding of this study is that hospital overcrowding and privacy concerns remain major barriers to allowing it (Munson et al. 2025; Sarwal et al. 2023; Singh et al. 2021; Yaya Bocoum et al. 2023). This finding is consistent with the research conducted by Doba et al. (2023), whose study of birth attendants’ attitudes and the practice of companionship in Ethiopia reveals that societal norms and cultural beliefs are barriers to practising companionship. These findings suggest that encouraging men to be birth companions in Namibia would present a significant challenge because of the deeply ingrained cultural practices and long-standing beliefs that have shaped local societal norms around childbirth (Wesson et al. 2018). There is thus a pressing need to conduct comprehensive awareness campaigns that educate both men and women about the benefits of male involvement during childbirth, as well as to challenge and change the stereotypes that hinder progress towards shared parental responsibilities in this vital aspect of family life.

Participants advocated for specific changes to the LCG to improve record-keeping and quality of care. They recommended adding a column for vaginal delivery type (spontaneous, induced or instrumental) and increasing column sizes. This aligns with findings by Vogel et al. (2021) that vital information like blood type and HIV status is often missing. No literature was found to support an oxytocin space, possibly because of cultural factors in the Kavango region. These findings are critical for healthcare workers, as including this information is essential for proper maternal care.

Despite major challenges relating to the use of the LCG, the midwives highlighted some positive experiences, such as the contribution of supportive care elements, such as oral fluids, posture and pain relief. The interviewees described how these components contribute to maternal hydration, pain relief during labour and facilitation of foetal descent, ultimately resulting in a favourable birth outcome for both the mother and baby. These findings align with the research conducted by Vogel et al. (2021), which demonstrated that practitioners emphasised the use of the LCG as a guide for providing supportive care, regardless of their familiarity with the WHO guidance on supportive care. However, midwives in this study were hesitant to administer analgesics regularly because of concerns regarding potential side effects on both the mother and baby; paracetamol was identified as the most frequently used pharmacological pain relief method in this study. In line with Laisser et al. (2021), some midwives in this study questioned the necessity of pain relief as outlined in the LCG, stating the side effects on the baby.

Clearly, midwives agree that LCG is an effective tool for monitoring labour and identifying potential issues. However, they suggest that the tool could be improved by adding more columns and a section to specify the type of vaginal delivery, such as spontaneous, induced or assisted. This study has underscored the LCG’s profound importance in facilitating the monitoring of labour, detecting abnormalities at an early stage, and enabling timely interventions to prevent complications, as previously highlighted in scholarly works (Pingray et al. 2021; Vogel et al. 2021).

### Strengths and limitations

The study’s strengths include the adoption of a qualitative exploratory design and providing rich data on the experiences and perceptions of midwives on the implementation of the LCG in Namibia, while ensuring a comprehensive documentation of the methods used by employing a COREQ qualitative checklist. These results may provide valuable insights for national health policies, including the achievement of SDG 3 on good health and well-being. However, the study had a few limitations. Future research should carefully consider the potential for emotional impact and alternative data collection methods that prioritise participant cultural norms for comfort and psychological safety.

## Conclusion

This study concluded that midwives have had both positive and negative experiences while utilising the WHO’s LCG. Some midwives have reported that using the guide allows them to monitor labour in real-time, ensuring the well-being of both the mother and her baby. The LCG also provides vital information about the mother’s condition and facilitates timely interventions, which may reduce labour complications and improve maternal and child outcomes. These findings could inform national health policies and contribute to achieving SDG 3 (good health and well-being) by 2030. Midwives also appreciate the additional care components that the LCG provides, such as pain relief, posture, and oral fluids. These elements enhance the childbirth experience and empower midwives to provide comprehensive care.

The use of traditional oxytocin (Sivatu) to speed up labour complicates midwives’ ability to monitor labour progression, according to the LCG. This traditional oxytocin can induce spontaneous delivery, causing a significant life-threatening risk for both the mother and infant because of intense and rapid contractions. To address this issue, it is vital to explore alternative methods and strategies that allow for safe labour monitoring and management, while minimising the risks associated with traditional oxytocin. Additionally, the omission of the latent phase of labour can pose a diagnostic challenge and hamper the timely identification of prolonged labour and other abnormalities. Moreover, a lack of essential equipment, such as foetal heart monitors, further impedes midwives’ ability to recognise potential risks, increasing healthcare costs and increasing the danger for both mother and child. Future research should prioritise conducting a comprehensive study on the acceptability, usability, and feasibility of the LCG using mixed methods research.
